# Mosquito abundance, bed net coverage and other factors associated with variations in sporozoite infectivity rates in four villages of rural Tanzania

**DOI:** 10.1186/1475-2875-7-59

**Published:** 2008-04-18

**Authors:** Eliningaya J Kweka, Watoky MM Nkya, Aneth M Mahande, Charles Assenga, Franklin W Mosha, Ester E Lyatuu, Charles P Massenga, Edwin M Nyale, Stephen B Mwakalinga, Asanterabi Lowassa

**Affiliations:** 1 Tropical Pesticides Research Institute, Division of Livestock and Human Disease Vector Control, P.O.Box 3024, Arusha, Tanzania; 2 Joint Malaria programme, P.O.Box 2228, Moshi, Tanzania; 3 Kilimanjaro Christian Medical Centre, P.O.Box 3010, Moshi, Tanzania; 4 Centre for Medical Parasitology, Copenhagen University, Denmark; 5 Tanzania Wildlife Research Institute, P.O.Box 661, Arusha, Tanzania

## Abstract

**Background:**

Entomological surveys are of great importance in decision-making processes regarding malaria control strategies because they help to identify associations between vector abundance both species-specific ecology and disease intervention factors associated with malaria transmission. Sporozoite infectivity rates, mosquito host blood meal source, bed net coverage and mosquito abundance were assessed in this study.

**Methodology:**

A longitudinal survey was conducted in four villages in two regions of Tanzania. Malaria vectors were sampled using the CDC light trap and pyrethrum spray catch methods. In each village, ten paired houses were selected for mosquitoes sampling. Sampling was done in fortnight case and study was undertaken for six months in both Kilimanjaro (Northern Tanzania) and Dodoma (Central Tanzania) regions.

**Results:**

A total of 6,883 mosquitoes were collected including: 5,628 (81.8%) *Anopheles arabiensis*, 1,100 (15.9%) *Culex quinquefasciatus*, 89 (1.4%) *Anopheles funestus*, and 66 (0.9%) *Anopheles gambiae s.s*. Of the total mosquitoes collected 3,861 were captured by CDC light trap and 3,022 by the pyrethrum spray catch method. The overall light trap: spray catch ratio was 1.3:1. Mosquito densities per room were 96.5 and 75.5 for light trap and pyrethrum spray catch respectively. Mosquito infectivity rates between villages that have high proportion of bed net owners and those without bed nets was significant (P < 0.001) and there was a significant difference in sporozoite rates between households with and without bed nets in these four villages (P < 0.001).

**Conclusion:**

Malaria remains a major problem in the study areas characterized as low transmission sites. Further studies are required to establish the annual entomological inoculation rates and to observe the annual parasitaemia dynamics in these communities. Outdoor mosquitoes collection should also be considered.

## Background

Malaria morbidity and mortality in Africa has increased due to an increase of resistance of chloroquine and sulphadoxine-pyrimethamine (SP), insecticidal resistance [1-3] and social economic status [4]. In Tanzania, malaria is the main cause of admission for children (38%) and adults (32%) in health facilities [5-7]. Estimates show that 28 million Tanzanians are exposed to the risk of stable malaria, resulting in 16 million clinical episodes per year and 100,000 child deaths over 25% of total deaths [8].

The levels of transmission of malaria as measured by the entomological inoculation rate are likely to be highly variable among sites in Africa including Tanzania [9]. Awareness of factors contributing to the intensity of malaria transmission over time is extremely important for choosing and targeting malaria control interventions in rural settings. Recent information on malaria transmission intensity in rural Tanzania are inadequate for prevention due to poor recording system within the communities [4].

Therefore, it was the objective of this study to assess the mosquito species composition and host blood meal origin, sporozoite infectivity rates, and bed net coverage within four villages with different ecological characteristics located in two regions of mainland Tanzania.

## Materials and methods

### Study site description

Four villages, three in the Kilimanjaro region (Rundugai in Hai district, Kisangara in Mwanga district and Ndungu in Same district) and one in the Dodoma region (Chamwino village in Chamwino district) were surveyed. Chamwino, Rundugai and Ndungu are sentinel sites for the monitoring of anti-malarial drug efficacy and were selected by the National Malaria Control Programme in the framework of the East African Network for Monitoring Anti-malarial Treatment (EANMAT). Such selection, based on past records and the associated ecological diversity, ensured adequate representation of the heterogeneity of malaria endemicity in Tanzania (Table 1). There were no vector control activities in these sites or in the neighbouring areas either before or during the entomological survey, except Ndungu village that in 1958 had performed aerial spray with Dieldrin.

**Table 1:** Description of the study villages, their geographical features and meteorological information.

**Study Sites**	**Rundugai**	**Kisangara**	**Ndungu**	**Chamwino**
**Altitude(M)**	750	920	1050	700
**Rainfalls (mm)-2004**	589	290	980	280
**Min.Temp (0C)**	24.7	26.7	22.1	26
**Max.Temp (0C)**	29.4	31.2	29	30.3
**Landscape**	Rural northern Tanzania; extensive swamps and savannah grassland	Peri urban-semi arid, northern Tanzania	Rural, northern Tanzania, bare hills, swamps and grassland.	Rural-central Tanzania, semi-arid.
**Ethnic groups and Economic activities**	Masai, Pare and Chagga, growing maize, vegetable, rice and keeping large groups of cattle.	Pare, mainly growing maize and have cattle heads.	Pare, mainly growing maize, banana trees, sugarcane, rice and beans. They have cattle heads around.	Gogo, Growing maize and ground nuts, they have large groups of cattle around their houses.
**Any Cattle kept in compound about 5 to 10 meters from sleeping house.**	285 (71.4%)	605 (84.1%)	1509 (72.5%)	702 (67.4%)
**Mosquito net in selected households.**	322 (81.0%)	520 (72.3%)	1797 (86.0%)	512 (49.1%)

### Mosquito sampling and identification

Mosquitoes were collected inside of selected houses fortnightly using CDC light traps and pyrethrum spray catch methodologies [10]. Morphological classification was used to group and identify adult anophelines mosquitoes [11]. All specimens were stored dry in properly labelled tubes and maintained with silica gel. Species identification of the *Anopheles gambiae *s.l. was performed using polymerase chain reaction (PCR) [12]. The *Anopheles funestus *complex was identified to species level using the multiplex PCR technique of Koekemoer *et al *[13]. The head and thorax of the identified specimens were placed into individual polypropylene microcentrifuge tubes (1.5 ml) and dried over silica gel until further processing. Quality control for species identification was ensured by using two separate teams for *An. gambiae *s.1. and *An. funestus *morphologic identification as well as PCR processing.

### Mosquito sporozoite prevalence

The involvement of each species in malaria transmission was assessed using an enzyme-linked immunosorbent assay (ELISA) for *Plasmodium falciparum *sporozoite detection [14]. Monoclonal antibody 2A10, directed against circumsporozoite protein of *P. falciparum *(produced by the New York University), was run on every ELISA test plate. Positive controls consisted of 100 pg and 10pg of a recombinant *P. falciparum *circumsporozoite protein. Mosquitoes from an insectary colony of *An. gambiae s.s *were used for negative controls. Mosquito samples were tested monthly and each head and thorax was treated singly. Each mosquito head and thorax was placed per well of Elisa plate. Plates were incubated overnight at 4°C temperature. In confirmatory tests, samples were considered positive for sporozoite antigens only if the 30 min absorbance values using 415 nanometres filter in the ELISA plate reader exceeded the mean plus four standard deviation of the eight negative control mosquitoes. All *An. gambiae *s.l. samples that tested positive by ELISA for *P. falciparum *circumsporozoite protein were identified by PCR for sibling species identification.

### Host blood meal identification

Blood fed mosquitoes collected from the pyrethrum spray catch method were processed for host blood meal identification using the precipitin test [15]. Mosquitoes were smeared onto 9 cm Whitman filter paper and the blood meal extracted from the filter paper following the precipitin ring test protocol by Bray *et al *[15] using the human anti-sera at a dilution of 1:10,000.

### Meteorological and ecological village data

Monthly meteorological data were obtained from the Tanzania Meteorological Agency (TMA) located approximately five to six kilometres to each village. Ecological (topographical) data were collected by observation of environmental feature (vegetations) available. Meteorological data analysed included rainfall totals in millimetres and the average minimum and maximum temperatures for the six-month study period

### Social economic status

A detailed questionnaire was used to assess the availability of mosquito nets, numbers and types of animals kept around the house, ethnicity characterization and main economic activities of selected households within each study village. Trained field workers administered questioners in all villages. Head of households were responsible informants in all villages.

### Data analysis

In data analysis, two non-parametric tests were used. In examining the difference among the villages in sporozoite rates, the Kruskal Wallis test was performed. The effect of bed net coverage within villages was evaluated using the nested Mann-Whitney U test. The sporozoite prevalence rate was calculated as the number of positive circumsporozoite protein samples in all anopheline species based on the total number of anopheline species tested in each village.

### Ethical consideration

The ethical clearance was obtained from the Kilimanjaro Christian Medical College of Tumaini university, Institutional Ethics Committee and the National Institute of Medical Research (NIMR) Ethics committee.

## Results

**Figure 1:**
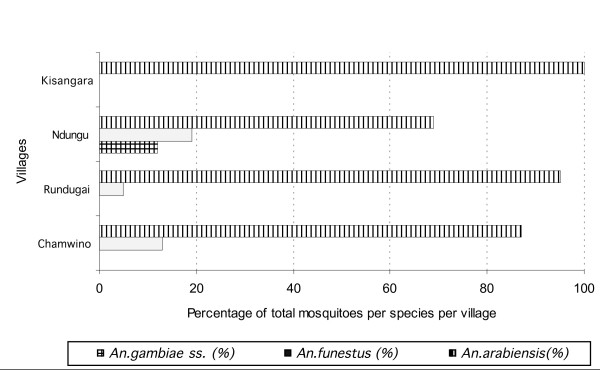
Prevalence of Anopheline mosquitoes in four villages, in two regions of mainland Tanzania.

A total of 6,883 anophelines were collected indoors from all selected houses during the six-month study period, *An. arabiensis *was the predominant vector in each of the four study villages. In Rundugai, *An. arabiensis *comprised 95% of the 2,001 total specimens, in Ndungu 69% of 3,164, Chamwino 87% of 1,340 and in Kisangara 100% of the 378 were *An. arabiensis *(Figure 1). In Ndungu village 12%, of 3,164 anophelines caught were *An. gambiae ss*. No *An. gambiae *ss were found in Kisangara, Rundugai, or Chamwino (Figure 1). *An. funestus *was found in three of the study sites: Ndungu (19% of 3,164), Chamwino (13% of 1,340), Rundugai (5% of 2,001), however no *An. funestus *were collected from Kisangara village (Figure 1).

### Sporozoite prevalence

**Figure 2:**
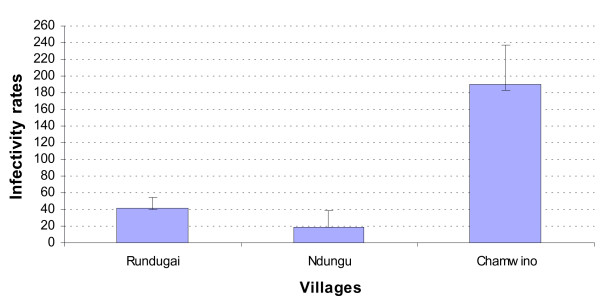
Sporozoite Infectivity rate means among the three study villages that had infected mosquitoes.

In total, 6,838 Anopheles mosquitoes of which 5,000 were *An. gambiae *s.1 and 1,838 *An. funestus *were tested for *P. falciparum *circumsporozoite protein infection by ELISA (Table 2). Transmission intensity was unstable in Rundugai, Ndungu and Chamwino while in Kisangara it was stable. There was a significant difference in monthly mosquito sporozoite prevalence rates between villages (Kruskal-Wallis χ^2 ^= 13.3, DF = 3, P = 0.004). Chamwino had the highest (5.3%), with Ndungu (1.3%) and Rundugai (1.6%) showing lowest sporozoite infectivity and no infected mosquito samples were collected from Kisangara during the study period. The monthly infectivity rates are shown in Figure 2.

**Table 2:** Sporozoite prevalence rates for Anopheles mosquitoes testes for circumsporozoite protein in each village monthly.

	Ndungu			Kisangara			Rundugai			Chamwino		
Months	SPR	SPM	TM	SPR	SPM	TM	SPR	SPM	TM	SPR	SPM	TM
April	0.3	1	310	0	0	210	3.8	14	361	10	28	280
May	1	4	410	0	0	206	2.7	3	110	0.9	1	109
June	0.3	1	290	0	0	173	0.2	1	413	8	34	421
July	0	0	150	0	0	359	1	4	390	2.9	11	370
August	1	5	509	0	0	198	0	0	348	0.9	3	301
September	5.1	10	194	0	0	104	2	5	241	9	34	382
Comparison of SPR in villages	χ^2 ^= 13.3 ; df = 3 and P = 0.004											

Key:

SPR = Sporozoite Prevalence rates; SPM = Sporozoite Positive mosquitoes; TM = Total Tested Mosquitoes

### Host blood meal identification

In total, 2,465 of the total collected mosquitoes were blood fed. The majority of *An. gambiae *s.l. 820 (85.7%) collected had obtained their blood meals from a human host with 135(14.3%) having neither bovine nor human bloodmeals (Table 3). Similarly, the majority of *Anopheles funestus *720 (88.7%) had taken a blood meal from human source with 92 (11.3%) having an undetectable blood meal host source. On the contrary, 608 (87.1%) of *Anopheles arabiensis *had a cattle blood meal, 80 (11.2%) had a blood meal from humans and 10 (1.4%) showed a mixed blood meal from both cattle and human (Table 3).

**Table 3:** Blood meal origins shown by different mosquito species caught indoors monthly in study villages.

**Host**	*An. gambiae*			*An. funestus*			*An. Arabiensis*			
	Sample Tested	Positive (%)	Negative	Total Tested	Positive (%)	Negative	Total tested	Positive samples (%)		

**Blood meal source**		**Human**			**Human**			**Cattle**	**Human**	**Mixed (human/cattle)**

April	120	104 (86.7%)	16	125	104 (83.2%)	21	117	98 (83.8%)	17 (14.5%)	2 (1.7%)
May	190	152 (80%)	38	109	95 (87.1%)	14	124	112(90.3%)	12 (9.7%)	0 (0%)
June	142	132 (92.9%)	10	114	94 (82.4%)	20	97	78 (80.4%)	16 (16.5%)	3 (3.1%)
July	163	126 (83.4%)	37	113	109 (96.5%)	4	142	130(91.6%)	8 (5.6%)	4 (2.8%)
August	90	79 (87.8%)	11	140	113 (80.7%)	27	68	51 (75%)	16 (23.5%)	1 (1.5%)
September	250	227 (90.8%)	23	211	205 (97.2%)	6	150	139(93.7%)	11 (6.3%)	0 (0%)

### Social economic status

The bed net coverage among surveyed households had a trend as Ndungu, Rundugai, Kisangara, and Chamwino is shown in Table 1, which ranged from 49.1% to 86.0%. There was significant difference in the groups with and without bed nets (Z= -3.740, P < 0.001). The large groups of animals around domiciles for about five to ten metres were observed in all villages (Table 1) which ranged between 50.2 % – 84.1 %. The groups of cattle around domiciles about five to ten metres have been associated with active malaria reduction [16-18]. Each village had its own agricultural activities that favoured by the weather in the locality (Table 1).

### Meteorological data

Monthly rainfall totals ranged from 280 mm to 980 mm during the study period in all villages in which April and May were highest rainfall season and lowest in August and September. Minimum temperature ranged from 22.1°C to 26.7°C while maximum temperature was between 29°C and 33.6°C. The highest temperatures were experienced in Chamwino village while the lowest was in Ndungu village with mountainous characteristics at an altitude of 1,050 m above sea level.

## Discussion

Previous studies indicated that *An. gambiae *s.s, *An. funestus *and *An. arabiensis *are major malaria vectors in Tanzania [19,20]. The objective of this study was to conduct entomological surveys in four ecological distinct villages to characterize the association of mosquito abundance of these main malaria vectors with sporozoite rates, host blood meal sources and bed net coverage. The sporozoite prevalence rate estimates the level of exposure to the malaria parasite-infected mosquitoes and is a commonly used index for assessing the malaria endemicity and the transmission rate. Weak ELISA reactions were excluded to avoid an over estimation of the sporozoite prevalence rate [14,21]. Even distribution of mosquitoes among houses of the same village is rare as adult wild mosquito populations often exist in clusters so that changing the houses sampled could have increased the chances of not capturing the seasonality of the sporozoite prevalence rate [22]. Therefore, in each sentinel site, to estimate the sporozoite prevalence rates and mosquitoes abundance sampling was done in the same twenty houses throughout the surveys, other houses were omitted from the study period due to the absence of house members during trapping days. There was no vector control activities done nearly before or during the study implementation, the estimated sporozoite prevalence rates of malaria vectors could be used as a reference for any vector control program in these villages. Important differences in infectivity rates were observed between the study sites, ranging from 0% infective rate found in Kisangara to 10% in Chamwino. In all villages *An. arabiensis *predominated. These villages had average sporozoite prevalence rates < 10%. There was observed monthly variation in infection rates for each village as shown in Table 2 and, therefore, can be classified as unstable malaria areas [23-25]. *Anopheles arabiensis *played an important role in malaria transmission, but not proportional to its relative abundance. It contributed highest proportion of the overall of the total anophelines collected, but only 18% of the tested specimens indicated sporozoite infection for Chamwino, Ndungu and Rundugai villages. Drier environments favour *An. arabiensis *and the adaptation of this species to peri-urban environments has been observed elsewhere in Africa [26]. However, in the rural Ndungu area along the Kalimawe swamp, *An. arabiensis *is dominant on the valley basin while *An. gambiae *s.s. is abundant on the down foothills [27]. Similar patterns were observed around the Lake Tanganyika, with predomination of *An. arabiensis *in the northern valley and *An. gambiae *s.s. along the southern foothills [28,29]. The observed sporozoites rate values for these four villages are in line with the past findings and recent predictions of malaria endemicity [23]. However, it is important to establish how the observed differences in infectivity rates in Kisangara, Chamwino, Ndungu and Rundugai translate into disease burden and what control effort is needed to decrease transmission in each village.

Results from blood meal identifications indicate that the presence of cattle within the peridomestic environment for about five to ten metres can serve as an alternative blood meal source when the community is well protected by ITNs and the predominate species is *An. arabiensis*. This data supports previous studies examining anopheline blood meal sources [16,18,30] that describe *An. arabiensis *to be more attracted to cattle than *An. gambiae *s.s. It is possible that the proper use of ITNs within a community increases the probability of mosquitoes to exit homes and hence cause animals to be an alternative blood meal source [18,31-33]. Likewise, the proper use of Indoor Residual Spray (IRS) may be better tool in controlling endophagic mosquitoes (*An. gambiae *s.s.,*An. funestus *and *An. arabiensis*) in areas were they are predominant species, such as described by Worrall *et al *[34] in Zimbabwe.

The insecticide-treated net (ITN) coverage is one of the key interventions for malaria control [6] in rural Tanzania. ITN coverage has a high probability of being effective in controlling malaria because of the endophagic behaviour of the three main vectors (*An. gambiae *s.s, *An. arabiensis *and *An. funestus*) in Tanzania [35]. The lower bed net coverage in Chamwino village (41%) has been speculated as the major factor for higher sporozoite prevalence rate due to easier access to a human blood meal. In Ndungu and Rundugai the bed net coverage was above 80% while in Kisangara the bed net coverage was 72% which may have provided better community protection [36]. Maxwell *et al *[37,38] found that large coverage of ITNs (75% and above) provides better protection to the whole community including those without bed nets. Findings from Asembo in Western Kenya indicated that most important reasons for non-adherence to use of ITNs was disruption of sleeping patterns due to visitors, funerals, house constructions and other events [39]. Another study in the same area showed that some people believed that ITNs would only be partly effective due to the perception that malaria has multiple causes [40]. Concerns included fear of the insecticide that is thought by some to be a toxic family planning aid [40]. The poor bed net coverage and malaria transmission in sub-Saharan Africa has been also contributed to poor economic growth rate of these countries [4,6]. The effectiveness of ITNs however, is not only measured by the coverage but also includes proper adherence of net use periodic re-treatment of the nets, as well as the vector species involved in the malaria transmission in the region or village [41-43]. In western Kenya, *An. funestus *was strongly affected by just the presence of at least one treated net within the house, and compliance seems to be less important when *An. funestus *is the predominant vector such as in Rundugai and Ndungu. Long-lasting insecticidal nets (LLINs) are now available and are the appropriate response for low re-treatment rates of conventional ITNs [44-46]. Educational activities should also play an integral role in malaria control strategies to assure good house design in preventing house entry of vectors and the proper use of ITNs.

## Conclusion

Control measures against infective mosquito bites have a major beneficial impact on malaria morbidity and mortality. Implemented vector control strategies should provide a community-wide health impact that will vary effectively between localities with consideration of high proportions of the disease vector abundance and transmission level. Malaria vector feeding and resting behaviour should be taken into consideration for initiation of malaria control strategies.

## Competing interests

The authors declare that they have no competing interests.

## Authors' contributions

EJK, FWM, SBM and WMMN conceived and designed the study, participated in analysis and interpretation of data and drafted and edited the manuscript. AL did analysis and interpretation of sociological (survey) data. EJK, AMM, CA, EEL, CPM, EMN participated in data collection in the field and laboratory analysis. All authors read and approved the final manuscript.
